# Effect of ultrasound parameters of benign thyroid nodules on radiofrequency ablation efficacy

**DOI:** 10.1186/s12880-023-01044-z

**Published:** 2023-06-19

**Authors:** Zahra Motaghed, Hossein Chegeni, Ali Mosadeghkhah, Mohammadreza Azimi Aval, Reza Gerami, Hojat Ebrahiminik

**Affiliations:** 1 Faculty of Medicine, AJA University of Medical Sciences, Tehran, Iran; 2 Tirad Imaging Institute, Tehran, Iran; 3 Department of Interventional Radiology and Radiation Sciences Research Center, AJA University of Medical Sciences, Tehran, Iran; 4 Department of Radiology, Faculty of Medicine, AJA University of Medical Sciences, Tehran, Iran

**Keywords:** Interventional Radiology, Radiofrequency Ablation, Thyroid Nodule, Treatment Outcome

## Abstract

**Background and aim:**

Ultrasound-guided radiofrequency ablation (RFA) is a minimally invasive therapy for thyroid nodules (TNs). Understanding the determinants of RFA efficacy can improve treatment and prognosis. This study aims to investigate the relationship between ultrasound parameters of benign TNs and the efficacy of RFA.

**Methods:**

A pretest–posttest interventional study was conducted in 2021 on 250 randomly sampled patients with benign TNs, receiving RFA. For this purpose, the volume reduction (VR) and the VR ratio (VRR) of the nodules were measured at the 1-, 3-, 6-, and 12-month follow-up periods after the RFA completion. The technical success rate (TSR) of this procedure was then categorized into four states, including low (VRR < 25%), moderate (VRR = 25–49%), high (VRR = 50–74%), and very high (VRR ≥ 75). Ordered logistic regression (OLR) was further utilized to investigate the effect of the ultrasound parameters of TNs on the TSR. The analyses were notably performed using Stata 14.2.

**Results:**

The VRR at the 1-, 3-, 6-, and 12-month follow-up periods were 38.7%, 53.6%, 59.3%, and 59.9%, respectively. The mean VR was also statistically significant at all follow-ups (*p* < 0.001). At the 1-, 3-, and 6-month follow-up periods, the VR of over 50% was observed in 28.2%, 52.1%, and 65.2% of the nodules, respectively. The odds ratios (ORs) of the RFA success were found to be 4.3 times higher for the nodules in the left lobe compared to the right lobe (OR: 4.31, *p* = 0.002), 6.3 times greater for isoechoic nodules compared to hyperechoic nodules (OR: 6.39, *p* < 0.001), 6.2 times higher for hyper-vascular nodules compared to hypo-vascular nodules (OR: 6.25, *p* = 0.005), and 2.3 times greater for mixed nodules compared to solid ones (OR: 2.37, *p* = 0.049).

**Conclusion:**

The ultrasound parameters of TNs had a statistically significant effect on the efficacy of RFA. Small-sized, isoechoic, and hyper-vascular nodules, as well as those with mixed tissue, were observed to respond better to RFA, leading to a better prognosis in terms of VR after treatment.

## Introduction

Thyroid nodules (TNs) are among the common diseases of the endocrine system [1]. According to the autopsy and ultrasound reports, roughly 40–50% of the world’s general population suffer from the abnormal overgrowths of tissue in the thyroid gland [2]. Such nodules can be identified in 4–7% of the general population through physical examinations, or via palpation in 1.5% and 6% of men and women, respectively [3, 4]. Autopsy and ultrasound reports also suggest that approximately 30–70% of TNs form in middle-aged people (namely, 50 years of age on average) and that they are more common in Asian populations [4].

Overgrown TNs tend to put much pressure on the surrounding tissue, causing discomfort and sensation of a foreign body in the throat, swallowing difficulties, hoarseness, shortness of breath, and external symptoms. If left untreated, these nodules may result in life-threatening conditions, such as acute respiratory arrest (ARA) [5]. Even if TNs are diagnosed as benign during a biopsy, there is a 6% probability to be malignant in a post-operative autopsy, as evidenced in previous research [6]. Therefore, it has been recommended to treat symptomatic benign TNs actively, as if they were potentially malignant, in order to prevent any potential life-threatening complications [7].

Considering the risks associated with surgery (even simple lobectomy) on TNs, including laryngeal paralysis, scarring, infection, compressive hematoma, hypocalcemia, and lifelong hormone replacement therapy (HRT) [8] for the benign cases, it is better to practice less invasive and expensive treatment methods. Radiofrequency ablation (RFA) has been accordingly recommended as the first-line therapy for some cases [9]. Despite the availability of guidelines for managing TNs, surgery is still excessively used as the first-choice treatment, which has become a challenge [10]. Along with the latest evidence in France, nearly 10,000 unnecessary thyroidectomies are done each year in patients with the most-likely benign nodules [11]. In one study, more than half of the operated TNs were thus found to be benign after surgery, indicating a trend of over-surgery on them [12].

Ultrasound-guided RFA is known as a minimally invasive non-surgical therapy that was first employed for the treatment of benign and advanced malignant liver tumors [13]. Considering the advantages of this procedure in terms of safety, efficacy, lack of incision and scarring, and absence of hormone therapy after completion, it is a viable choice to treat benign TNs and even hard-to-operate malignant ones [14, 15]. Given the relatively short history of the application of RFA in the treatment of TNs, many questions still remain regarding the determinants of its efficacy [16].

To improve RFA efficacy and minimize its side effects, it is crucial to shed light on the factors that may affect how well this procedure can deliver the planned results. As evidenced in the related literature, RFA tends to reduce the volume of TNs and alleviate their symptoms over time [17]. However, to the best of the authors’ knowledge, the present study is the first attempt to investigate the effect of ultrasound parameters of TNs (including tissue condition, echogenicity, vascularity, etc.) on its efficacy. The efficacy of RFA was examined in this study to determine whether and how much it was influenced by the ultrasound parameters of TNs.

## Methods

This interventional study was conducted in 2021 on a population of randomly selected Iranian patients with benign TNs who received RFA. Using the sample size formula for studies with a pretest–posttest design with a 5% error and 95% confidence interval (CI) for an expected effect size of 37% based on previous studies [5], the minimum sample size was determined to be 96 cases. However, it was decided to recruit a sample size of 250 to improve the accuracy of the findings and account for the possibility of dropouts. The random selection process was conducted among the pool of eligible patients referred by an Endocrinology specialist, and each eligible patient had an equal chance of being chosen. The selection was not based on any specific characteristics such as patient age, sex, or nodule size, but rather on the patient’s eligibility for RFA treatment, which was determined by the Endocrinology specialist based on the patient’s clinical condition and the characteristics of the thyroid nodules. Out of the 250 patients, 66 cases were excluded because they did not return in time for the 1-, 3-, 6-, and 12-month follow-up periods. Therefore, data from 184 patients were ultimately collected and analyzed. The participation and dropout rates were also equal to 74% and 26%, respectively.

$$n=\frac{{\left({Z}_{1-a/2}+{Z}_{1-\beta }\right)}^{2}}{{\Delta }_{plan}^{2}}+\frac{{Z}_{1-a/2}^{2}}{4}=(12.955/0.1369)+(0.9604)=95.5\sim 96$$

$$\mathrm\alpha=\mathrm{Probability}\;\mathrm{of}\;\mathrm{type}\;\mathrm I\;\mathrm{error}\;(5\%)/\beta=\mathrm{Probability}\;\mathrm{of}\;\mathrm{type}\;\mathrm{II}\;\mathrm{error}\;(5\%)/\Delta_{\mathrm{plan}}=\mathrm{Expected}\;\mathrm{standardized}\;\mathrm{effect}\;\mathrm{size}\;(37\%)$$

**Fig. 1:**
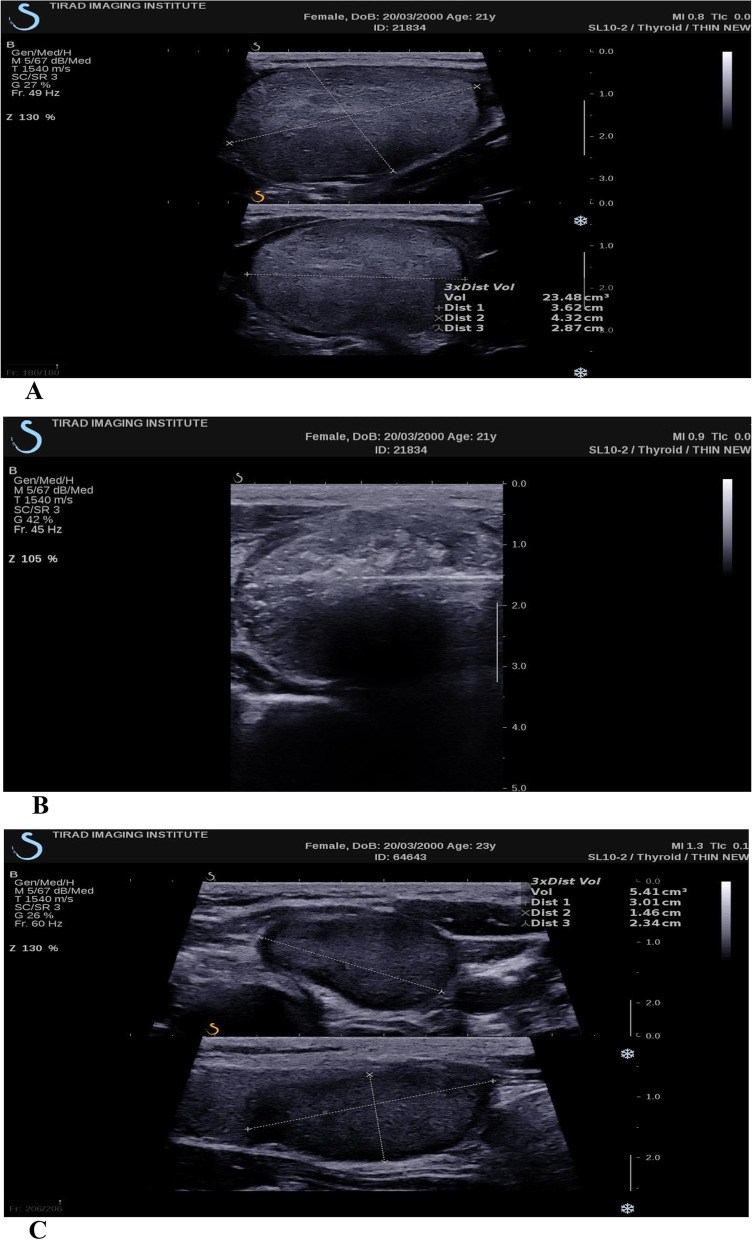
**A** Thyroid nodule size before RFA treatment, **B** RFA procedure on thyroid nodule, and **C** Post-RFA, 12-month follow-up of thyroid nodule
- Stage 2: The patients underwent RFA, wherein an RFA System was used to emit radio waves proportional to the nodule size according to the protocol [18], causing the destruction of nodule cells through friction and heat transfer. The data collected at this stage included the date and description of the RFA procedure. It is worth noting that an interventional radiologist with 7 years of experience performed the RFA treatment and was assisted by one radiology resident who provided support during the treatment under the guidance of the experienced radiologist.
- Stage 3: The change in nodule size after RFA was measured by ultrasound during the 1-, 3-, 6-, and 12-month follow-up periods. To minimize the impact of the measurement error on the findings, all RFA procedures and the nodule size pre- and post-measurements were completed by the same interventional radiologist, using the same machines and the same method.

The inclusion criteria for this study were as follows: age of at least 18 years old, diagnosis of benign TNs confirmed by two-time fine needle aspiration (FNA) biopsies, euthyroidism, normal profile of thyroid hormones in blood tests, and informed consent. Patients for whom TN treatment was not indicated, such as those who did not experience swallowing difficulties, shortness of breath, hoarseness, sensation of a foreign body in the throat, or dissatisfaction with their appearance, were excluded from the study. Additionally, patients with hypothyroidism and hyperthyroidism were also excluded. The data collection was performed in three stages:

- Stage 1: The demographic characteristics of the patients (i.e., age and gender) and the ultrasound parameters of the TNs (i.e., nodule size in diameter, tissue condition, echogenicity, vascularity, and location) were collected from the diagnostic imaging and pathological reports, along with the ultrasound results. The nodule volume was automatically calculated in cm^3 (equivalent to milliliters, ml) by the ultrasound device using the ellipsoid formula based on the measurements of the nodule’s three dimensions (length, width, and height). These measurements were taken by experienced radiologists using standardized protocols to ensure consistency and accuracy. Moreover, the measurements were obtained in multiple planes to ensure accuracy and minimize error. The before-after treatment images are presented in the Fig. 1.

In the descriptive phase of the study, the mean nodule volume reduction (VR) in ml and the VR ratio (VRR) in percentage (%) at the 1-, 3-, 6-, and 12-month follow-ups were calculated. The technical success situation (TSS) and the technical success rate (TSR) were further measured and reported as the measures of the RFA efficacy. Following the approach taken in previous studies [19], the TSS was defined as an over 50% reduction in the nodule size. Accordingly, two states were outlined for the TSS, viz., success (over 50% VR) and failure (below 50% VR). Moreover, the TSR was quartered at four states, namely, low (below 25% VR), moderate (25–49% VR), high (50–74% VR), and very high (75–100% VR).

In the analytical phase of the study, the comparison of the means was used to examine the RFA efficacy by checking whether the nodule size had significantly changed after the procedure. Since the Shapiro–Wilk test outcomes rejected the normality of the data, the non-parametric Wilcoxon signed-rank test was utilized to compare the mean nodule sizes before and after RFA. Finally, the ordered logistic regression (OLR) was employed to determine the effect of the ultrasound parameters of TNs on the TSR. As well, the TSR was introduced into the regression model as a qualitative ordinal variable (with low, moderate, high, and very high success) and the effect of the ultrasound parameters on this variable was investigated. All data analyses were performed using Stata 14.2.

This study was conducted in compliance with all the principles of professional and scientific ethics along with the full observance of the confidentiality of patient information. Informed consent was further obtained from all participants. All experimental protocols were additionally approved by the Iran National Committee for Ethics in Biomedical Research with the code no. IR.AJAUMS.REC.1400.071, ensuring adherence to the guidelines set forth by this committee. Furthermore, the study followed relevant international clinical practice guidelines for radiofrequency ablation of benign thyroid nodules, to ensure the ethical conduct of the research and protection of human subjects.

## Results

The mean VR (in ml) and VRR (in %) at the 1-, 3-, 6-, and 12-month follow-up periods are presented in Table 1. One month after RFA, a decrease in the size of all nodules was observed, with a mean VR of 17.6 ml (38.7% VRR). Additionally, the mean VR in the 3-, 6-, and 12-month follow-ups was 24.5 ml (53.6% VRR), 27.8 ml (59.3% VRR), and 27.8 ml (59.9% VRR), respectively. The median of VRR was 36.1%, 54.2%, 61%, and 61.3% at 1, 3, 6, and 12 months after RFA, respectively. Regarding the marginal changes in VRR (i.e., changes in the current follow-up VR compared with the previous one), the results indicated that the greatest VRR occurred during the first month (with an average of 38%), and the VRR decreased over time. Specifically, the nodule volume decreased by only 14% on average from the first to the third month and by 5% on average from the third to the sixth month. After the sixth month, the volume change was not significant, and the marginal change in VRR for the 12-month follow-up period (compared with the 6-month follow-up) was below 1% (0.53%).

**Table 1:** Volume reduction of thyroid nodules after RFA

**Variable**	**Follow-up**	**Median**	**Mean**	** ± SD**	**Min**	**Max**	**Marginal change**^**a**^
**Volume Reduction (VR)**	1-month	12	17.67	19.5	0	110	17.67
	3-month	18	24.57	23.9	1.2	125	6.9
	6-month	19.95	27.80	27.2	1.2	159	3.23
	12-month	19.95	27.87	27.1	1.2	159	0.07
**Volume Reduction Ratio (VRR)**	1-month	36.18	38.75	18.6	0	89.2	38.75
	3-month	54.24	53.65	18.1	7.5	100	14.9
	6-month	61.01	59.38	17.6	20	100	5.73
	12-month	61.36	59.91	18.2	20	100	0.53

^a^Indicates the mean volume change from the previous measurement/follow-up

The mean sizes of TNs before and after the RFA procedure are compared in Table 2. In this line, the TN size reductions at all follow-ups were found to be statistically significant (*p* < 0.001).

**Table 2:** Comparison of mean nodule sizes before and after RFA

**Variable**	**Median**	**Mean**	**S.E**	**95% confidence interval**		**Z**	***P*-Value**
				**Lower**	**Upper**		
Initial nodule size	40	49.98	3.75	42.58	57.38	-	-
Nodule size 1 month after RFA	27	32.30	2.48	27.39	37.21	11.758	0.0000
Nodule size 3 months after RFA	17	25.40	2.16	21.13	29.67	11.764	0.0000
Nodule size 6 months after RFA	14	22.18	1.97	18.28	26.08	11.764	0.0000
Nodule size 12 months after RFA	12.5	22.10	1.99	18.17	26.03	11.764	0.0000

**Fig. 2:**
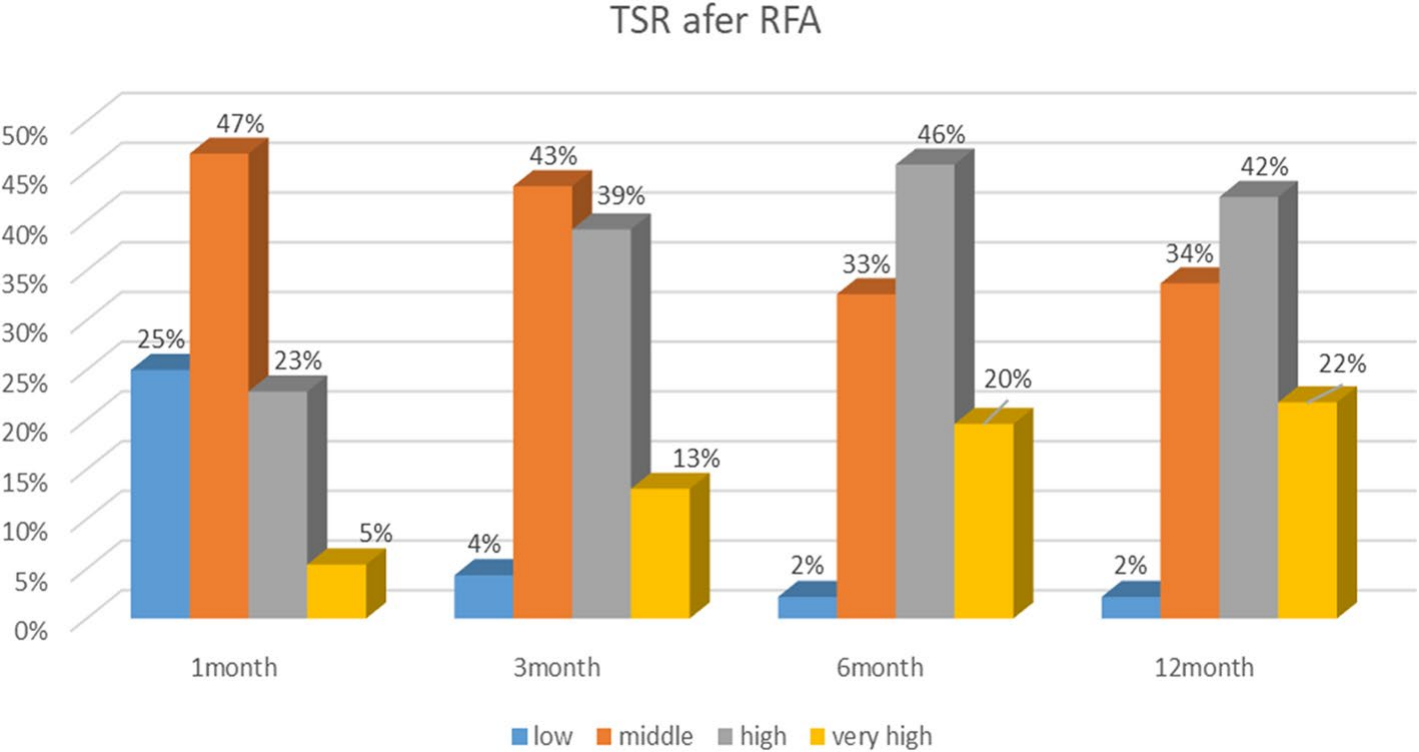
TSR of RFA at 1- to 12-month follow-ups

The TSS and TSR results are reported in Table 3 and plotted in Fig. 2. Based on the findings in Table 3, it was observed that the TSS of RFA (VRR > 50%) improved over time, increasing from 28.2% at the 1-month follow-up to 52.1% at the 3-month period, 65.2% at the 6-month follow-up, and finally settling at 64% one year after the RFA completion.

**Table 3:** Results of RFA efficacy measures

Variable	Follow-up	Success state	Frequency	Percentage
Technical Success Situation (TSS)	1-month	Success	52	28.26
		Failure	132	71.74
	3-month	Success	96	52.17
		Failure	88	47.83
	6-month	Success	120	65.22
		Failure	64	34.78
	12-month	Success	118	64.13
		Failure	66	35.87
Technical Success Rate (TSR)	1-month	Low	46	25
		Moderate	86	46.74
		High	42	22.83
		Very High	10	5.43
	3-month	Low	8	4.35
		Moderate	80	43.48
		High	72	39.13
		Very High	24	13.04
	6-month	Low	4	2.17
		Moderate	60	32.61
		High	84	45.65
		Very High	36	19.57
	12-month	Low	4	2.17
		Moderate	62	33.70
		High	78	42.39
		Very High	40	21.74

The TSR results presented in Fig. 2 illustrate that during the first month after RFA, low success was observed for 25% of the nodules, but this value decreased to 2% at the 6-month follow-up. Additionally, RFA was observed to be highly successful (VRR > 75%) for only 5% of the nodules at the 1-month follow-up, but this value increased to 21% at the 12-month follow-up (Fig. 2).

The OLR analysis in Table 4 examined the impact of ultrasound parameters of TNs on the TSR, and the results suggest that, holding other factors constant:

- RFA is three times more likely to succeed on small-sized nodules than large-sized nodules (OR: 3.02, *p* = 0.004).
- RFA is two times more likely to succeed on left-lobe nodules than right-lobe nodules (OR: 2.03, *p* = 0.031).
- RFA is 5.3 times more likely to succeed on hypoechoic nodules than hyperechoic nodules (OR: 5.36, *p* = 0.004).
- RFA is 3.2 times more likely to succeed on isoechoic nodules than hyperechoic nodules (OR: 3.26, *p* = 0.003).
- RFA is 4.1 times more likely to succeed on hyper-vascular nodules than hypo-vascular nodules (OR: 4.14, *p* = 0.002).
- RFA is 6.6 times more likely to succeed on mixed nodules than cystic nodules (OR: 6.66, *p* = 0.004).
- RFA is 2.4 times more likely to succeed on mixed nodules than solid nodules (OR: 2.45, *p* = 0.006).

**Table 4:** Effect of ultrasound parameters of TNs on TSR of RFA

Variable	Group	Odds Ratio (OR)	Standard Error (SE)	Z-value	*P*-value	95% confidence interval	
						Lower	Upper
**Age group**	Young (18–44)	1.406077	0.69536	0.69	0.491	0.5334046	3.706479
	Middle-aged (45–59)	1.843387	0.9301306	1.21	0.225	0.6856777	4.955793
	Elder (≥ 60)	Reference group	Reference group	Reference group	Reference group	Reference group	Reference group
**Gender**	Male	2.839874	1.123343	2.64	0.008	1.307968	6.165967
	Female	Reference group	Reference group	Reference group	Reference group	Reference group	Reference group
**Nodule size**	Small (< 15 ml)	3.020012	1.144465	2.92	0.004	1.436935	6.347174
	Medium (15–30 ml)	0.9743401	0.3984701	-0.06	0.949	0.437119	2.171808
	Large (> 30 ml)	Reference group	Reference group	Reference group	Reference group	Reference group	Reference group
**Nodule location**	Right lobe	Reference group	Reference group	Reference group	Reference group	Reference group	Reference group
	Left lobe	2.036833	0.6714487	2.16	0.031	1.067466	3.886483
	Isthmus	1.955425	0.9727208	1.35	0.178	0.7375868	5.184048
**Nodule echogenicity**	Isoechoic	3.26388	1.284177	3.01	0.003	1.509482	7.05733
	Hypoechoic	5.363904	3.15472	2.86	0.004	1.693777	16.98657
	Hyperechoic	Reference group	Reference group	Reference group	Reference group	Reference group	Reference group
	Anechoic	3.228531	3.866898	0.98	0.328	0.3086728	33.76849
**Nodule vascularity**	Iso-vascular	1.582261	0.5854298	1.24	0.215	0.7661916	3.267526
	Hypo-vascular	Reference group	Reference group	Reference group	Reference group	Reference group	Reference group
	Hyper-vascular	4.149143	1.907493	3.10	0.002	1.685132	10.21605
**Nodule tissue type**	Solid	0.406981	0.134009	2.73-	0.006	0.2134489	0.7759866
	Cystic^a^	0.1499492	0.0976556	2.91-	0.004	0.04184	0.5373981
	mixed	Reference group	Reference group	Reference group	Reference group	Reference group	Reference group
Regression fitness indicators							
** Psudue (R**^**2**^**)**				**0.1504**			
** Prob.chi2**				**0.0000**			
** Log-likelihood**				**-179.04787**			

^a^Almost completely cystic TNs with mural nodule

The results also demonstrated a statistically significant relationship between gender and the RFA success. Assuming that other factors were constant, RFA was 2.8 times more likely to be successful in men than in women (OR: 2.83, *p* = 0.008).

## Discussion

In this study, the mean and median of the nodule VRR one year after RFA completion were 59.9% and 61.3%, respectively. The TSS of RFA at the 12-month follow-up was also 64%, implying that 12 months after this procedure, 64% of the nodules had more than 50% VR. The first report on the clinical use of RFA on benign TNs in Iran was published by Ebrahiminik et al. [20] in the Iranian Journal of Radiology in 2017. They successfully treated 50 patients with 63 solid or cystic nodules with RFA, achieving 40–67% VRR at 1- and 3-month follow-up periods. In a similar investigation in 2021 by Deandrea et al. [18] on 115 Italian patients with benign TNs, the median VRR at the 12-month follow-up was 64% and the TSS was 75%. It was also described that one year after RFA, many patients, even those with below 50% VRR, no longer had compressive and cosmetic symptoms.

In the present study, the mean nodule VRR in the 1-, 3-, 6-, and 12-month follow-ups after RFA was 38.7%, 53.6%, 59.3%, and 59.9%, respectively, and their corresponding medians were 36.1%, 54.2%, 61%, and 61.3%, in that order. In Bisceglia et al. [21], examining 119 patients with benign TNs with a 48-month follow-up from 2014 to 2018, the mean VRR was 47.1%, 55.3%, 61.2%, and 67.7% in the 1-, 3-, 6-, and 12-month follow-up periods, respectively. A significant progressive improvement was further reported in VRR in the first two years after RFA up to the 24-month follow-up. In the present study, however, the greatest VR took place at the 1-month follow-up and its ratio dwindled over time, ultimately dropping to 0.53% at the 12-month period compared with the 6-month one. In this respect, Kuo et al. [22], investigating 109 nodules in 93 patients, found that the mean VRR was 55.9% six months after RFA. AAs well, Nguyen et al. [23], in a study on 78 benign TNs correspondingly reported the mean VRR of 41.4% and 64.7% and the TSR (VRR > 50%) of 30.8% and 84.6% at the 1- and 3-month follow-ups, respectively.

The results of the present study additionally established that the location of TNs could influence the success of the RFA procedure. More specifically, RFA was 2 times more likely to be successful on the left-lobe nodules than the right-lobe ones (OR: 2.03, *p* = 0.031). In Kuo et al. [22], reflecting on 102 nodules in 93 patients, VRR was further higher in the left-lobe nodules than the right-lobe ones, but this difference was not statistically significant. Similarly, Lee et al. [7] reported lower success (VRR > 50%) in the right-lobe nodules than the left-lobe ones, but there was no statistically significant difference in this respect (OR: 0.820, *p* = 0.557). The uneven efficacy of RFA in the left and right lobes could be thus related to the dissimilarities of the lobes in terms of anatomy and also the interventionist’s left- or right-handedness that need to be further examined in future studies.

Besides, the study results demonstrated a significant relationship between the RFA success and the echogenicity of TNs. Specifically, RFA was 5.3 and 3.2 times more likely to be successful on the hypoechoic and isoechoic nodules than the hyperechoic ones (OR: 5.36, *p* = 0.004; OR: 3.26, *p* = 0.003), which was consistent with the findings reported by Kuo et al. [24], suggesting a significant negative correlation between echogenicity and VRR in the solid nodules, in the sense that the success rate of RFA diminished as the nodule echogenicity elevated.

In this study, the RFA success was also found to be influenced by the vascularity of TNs. In particular, RFA was 4.1 times more likely to be successful on the hyper-vascular nodules than the hypo-vascular ones (OR: 4.14, *p* = 0.002). In the recent study by Deandrea et al. [18] on 337 patients in Italy, a significant relationship was similarly observed between the vascularity of TNs and the success of RFA. As well, the hyper-vascular TNs (i.e., those with intense peripheral and intranodal patterns) experienced a greater VR than the hypo-vascular and iso-vascular ones. Besides, a 71% reduction was reported in the hyper-vascular nodules as compared with the values of 68% in the hyper-vascular nodules and 67% in the iso-vascular ones; a difference that was statistically significant (*p* < 0.03). The reason for the discrepancy in the effectiveness of therapy for the hyper-vascular nodules could be thus explained by the creation of more coagulative necrosis in the hyper-vascular TNs after RFA and the resulting cell death, which could produce more VR in the nodules. In contrast to these findings, Kim et al. [25] in a study in 2006 on 35 nodules reported that vascularity had no significant impact on treatment response (*p* < 0.05).

The results of the present study confirmed that the RFA success was significantly correlated with the tissue condition of TNs, in the sense that RFA was 6.6 and 2.4 times more likely to be successful on the mixed nodules than the cystic and solid ones, respectively (OR: 6.66, *p* = 0.004; OR: 2.45, *p* = 0.006). This relationship could be thus attributed to the lower cellularity of the mixed nodules. The article published in 2006 by Kim et al. [25], as the first attempt on the clinical use of RFA to treat TNs, also reported that the mixed nodules responded significantly better than the solid ones (*p* < 0.05). Bisceglia et al. [21], examining benign TNs with a long-term follow-up from 2014 to 2018, further observed a significant positive relationship between treatment success (VRR ≥ 75%) and the presence of macrocystic structures (hazard ratio [HR] = 2.48, *p* = 0.046). As well, Kuo et al. [24] reported a significant relationship between the tissue condition of TNs and the success rate of RFA; namely, the efficacy of this procedure reduced with the higher proportion of the cystic components in nodules. In a study by Aysan et al. in 2016 [2], wherein one-session RFA significantly decreased (*p* < 0.001) the size of benign TNs from 16.8 ml before this procedure to 4.8 ml at the 3-month follow-up and 2.6 ml at the 6-month follow-up, the size reduction was greater in the cystic nodules than the solid and mixed ones. Deandrea et al. [18], investigating 337 patients in Italy also reported a significant relationship between the tissue condition of TNs and the success rate of RFA. As well, the spongiform TNs experienced a statistically significant (*p* < 0.01) size reduction (76%) than the solid (66%) and mixed (67%) ones. Likewise, Kuo et al. [22], reflecting on the success factors leading to RFA based on multivariate regression analysis on the data gathered from 109 nodules in 93 patients, showed significantly higher VRR in the cystic nodules than the solid ones (*p* = 0.04).

In the present study, RFA was 3 times more likely to be successful on the small-sized nodules than the large-sized ones (OR: 3.02, *p* = 0.004). Similarly, Deandrea et al. [18] reported that the RFA efficacy was negatively correlated with the nodule size, viz., the smaller the nodule, the greater the efficacy, and positively correlated with the amount of energy radiated to the nodule (per ml of the nodule volume). In Bisceglia et al. [21], the large initial nodule size (> 22.4 ml) based on the 4-year follow-up from 2014 to 2018 was found to be a predictor of the failure to achieve a VRR ≥ 75% after RFA (HR = 0.54, *p* = 0.036). In other words, the nodules with an initial volume of ≤ 22.4 ml responded significantly better to the treatment (*p* = 0.01). Likewise, Deandrea et al. [18] identified a weak inverse correlation between the initial nodule size and the nodule size reduction ratio (Spearman: -0.23). Feroci et al. [26], recruiting a small sample in Italy, also found that the nodules with an initial size of less than 20 ml had a greater VRR than those larger than 20 ml, and this difference was statistically significant at the 3- and 6-month follow-ups (*p* < 0.05), but not at the 12-month period (*p* = 0.167). In this respect, Lee et al. [7] also reported that RFA was 1.8 times more likely to succeed (VRR > 50%) on the nodules smaller than 4 ml than those larger than 4 ml (OR: 1.821, *p* = 0.032).

The present study correspondingly detected a statistically significant relationship between gender and the RFA success, in the sense that RFA was 2.8 times more likely to be successful in men than women, considering other factors being constant (OR: 2.83, *p* = 0.008). According to Lee et al. [7], investigating the determinants (VRR > 50%) of the RFA success on 1619 TNs, RFA was 1.2 times more successful in men than women, but this difference was not statistically significant (OR: 1.427, *p* = 0.430). Nguyen et al. [23], studying 78 benign TNs via multivariate regression analysis showed no statistically significant relationship between VRR and patient age (β: 0.29, *p* = 0.135) or gender (β: -16.09, *p* = 0.17). Considering the discrepancy in these findings, future studies are recommended to explore the relationship between the RFA efficacy and the level of sex hormones.

Despite the multitude of studies conducted on the efficacy of RFA in the treatment of benign TNs, little research has thus far investigated the effect of the ultrasound parameters of nodules on the efficacy of this procedure by multivariate regression analysis. In fact, most studies in this field have employed univariate analyses without respecting the effect of the confounding variables. The present study was accordingly the first attempt to perform a multivariate regression analysis with an OLR model to identify the effect of the ultrasound parameters of TNs on the RFA efficacy. Nevertheless, this study had two major limitations. First, almost all (95.6%) nodules examined in this study were non-calcified, which made it statistically impossible to investigate the effect of nodule calcification on the efficacy of RFA. The second limitation was the difficulty of maintaining a larger sample because of the patients’ poor commitment to follow-up schedules (here, the 1-, 3-, 6-, and 12-month follow-up periods), which caused 66 patients to be excluded from the study (of note, the participation rate was 74% and the sample dropout rate was 26%).

## Conclusion

The study results suggest that the ultrasound parameters of TNs may have a statistically significant effect on the efficacy of RFA. The small-sized, isoechoic, hyper-vascular nodules, and those with mixed tissue were found to respond better to RFA, resulting in a better prognosis in terms of VR. Therefore, reserving RFA for the appropriate cases and considering alternative treatments for cases where it is unlikely to be effective could improve the efficacy of RFA. The study findings could be helpful in reducing unnecessary and ineffective RFA procedures.

## Data Availability

The datasets generated during the study are available from the corresponding author upon reasonable request.
